# Risk factors for Low Anterior Resection Syndrome (LARS) in patients undergoing laparoscopic surgery for rectal cancer

**DOI:** 10.1007/s00464-021-09002-y

**Published:** 2022-02-08

**Authors:** Antonella Nicotera, Ezio Falletto, Alberto Arezzo, Massimiliano Mistrangelo, Roberto Passera, Mario Morino

**Affiliations:** 1 Department of Surgical Sciences, University of Torino, Corso Dogliotti 14, 10126 Torino, Italy; 2 Department of Medical Sciences, University of Torino, Torino, Italy

**Keywords:** Rectal-cancer, TME, Laparoscopy, LARS, Radiotherapy, Tumor-height

## Abstract

**Background:**

Total mesorectal excision (TME) represents the “gold standard” of rectal cancer surgery. In locally advanced lesions neoadjuvant treatments (e.g. radiotherapy-nRT, radio chemotherapy-cnRT) have been shown to improve TME oncological results, reducing local recurrences rate. Nevertheless, these treatments have significant functional consequences impacting patients’ quality of life (QoL). The resulting syndrome is known as Low Anterior Resection Syndrome (LARS). The purpose of this work was to evaluate the association between risk factors and the development of LARS in a prospective series of laparoscopic sphincter-saving TME.

**Methods:**

The study was conducted as a retrospective observational epidemiological study of a prospective database, including all patients undergoing laparoscopic anterior resection surgery for rectal cancer at our Unit from 1st January 2013 to 31st May 2018. The diagnosis of LARS was performed using the LARS Score. We classified risk factors in patient-related, pre-, intra- and post-operative factors.

**Results:**

The sample included 153 consecutive patients. Forty-one were affected by “low” rectal cancer, 74 by “middle” rectal cancer, 38 by “high” rectal cancer. The prevalence of overall LARS (major LARS + minor LARS) in our series was 35.9% (55/153 cases). Association between nRT and overall/major LARS was significant (respectively *p* = 0.03 and 0.02). Distal localization of tumor was also significantly associated with LARS [overall LARS (*p* = 0.03), major LARS (*p* = 0.014)].

**Conclusions:**

In our study, neoadjuvant radiotherapy and tumor localization resulted independent risk factors for LARS after laparoscopic sphincter-saving TME. Tumor localization in the “middle” and “high” rectum resulted a protective factor compared to the localization in “low” rectum.

Total mesorectal excision (TME) introduced by Heald in 1983 represents the “gold standard” of rectal cancer surgery [1]. When technically feasible and oncologically safe, surgeons strive to reconstruct intestinal continuity by sphincter saving techniques, progressively limiting in recent years the rate of abdominoperineal resection. In locally advanced lesions neoadjuvant treatments (e.g. radiotherapy, radio chemotherapy) have been shown to improve TME oncological results, reducing local recurrences rate [2–5]. Regrettably, the abovementioned treatments, although very effective from the oncological point of view, are associated with functional consequences significantly impacting patients’ quality of life (QoL). The resulting syndrome is known as Low Anterior Resection Syndrome (LARS). In 2012, Briant proposed a universally accepted definition of LARS: “Disordered bowel function after rectal resection, leading to a detriment in quality of life” [6]. LARS includes a variety of symptoms related to bowel dysfunction, such as defecation urgency, gas and stool incontinence, stool fragmentation, and obstructed defecation. Such symptoms are time-correlated, generally regressing in a variable interval from 6 to 18 months, time necessary for the neo-rectum to “rehabilitate” and time beyond which further improvements, if not occurred, become unlikely and the syndrome is considered irreversible. LARS is a complex syndrome, resulting from a multifactorial genesis. Best known LARS risk factors are lesion of the anal sphincter [7–10], TME and height of the anastomosis [11–15], neoadjuvant radiotherapy (nRT) [13–17], type of anastomosis and configuration of the neorectum [18], motility of the neorectum [19, 20], presence of diverting stoma and timing of its closure [21]. Currently, several scores are available for the diagnosis and classification of LARS. The most used is the LARS score, according to which the syndrome is divided into minor LARS and major LARS [22]. As well as the etiopathogenesis, the treatment is also multifactorial and in most cases symptom-based. Treatment includes anti-diarrheal drugs, anal plugs, biofeedback therapy, pelvic floor rehabilitation, colon irrigation and nerve stimulation [23].

The advent of laparoscopic surgery in the treatment of rectal cancer has improved the postoperative course, reducing both hospital stay and time to resume daily activities. Nevertheless, data on function outcome of the laparoscopic rectal resections are scarce and specific data on LARS are lacking. The purpose of this work was to evaluate the association between anamnestic, pre-, intra- and post-operative risk factors and the development of LARS in a prospective series of exclusively laparoscopic sphincter-saving TME.

## Materials and methods

### Study design

The study was conducted as a retrospective observational epidemiological study of a prospective database, including all patients undergoing laparoscopic anterior resection surgery for rectal cancer at our Unit from 1st January 2013 to 31st May 2018. The diagnosis of LARS was performed using the LARS Score, resulting from a questionnaire which consists of 5 questions, whose answers are associated with a score, the sum resulting in the final score. A score from 0 to 20 indicates absence of LARS, 20 to 29 minor LARS, 30 to 42 major LARS [22]. The Score was calculated through the reports of post-operative follow-up surgical visits or through a telephone interview, after checking the presence of informed consent to the processing of personal data for purpose of scientific research. The calculated LARS score referred to the first 12–18 months after surgery for each patient. The study was conducted in good clinical practice according to the Helsinki Declaration of 1975 and subsequent modifications.

### Aim of the study

The aim of the study is to estimate the prevalence of LARS in our Center and to define LARS risk factors in patients undergoing laparoscopic surgery for rectal cancer.

### Exclusion criteria

Patients undergoing laparoscopic surgery converted to open technique, patients who did not give their consent, patients who developed post-operative complications requiring a permanent stoma, deceased patients whose post-operative course could not be reconstructed and patients lost to follow-up were excluded.

### Population risk factors

We distinguished between patient-related risk factors and pre-, intra- and post-operative factors. Patient-related risk factors are: gender, age > 65 years, smoking, body mass index (BMI), cardiovascular disease (arterial hypertension, coronary heart disease, cardiomyopathies, electrical disorders), diabetes, neurological/neurodegenerative disorders, chronic obstructive pulmonary disease (COPD), chronic corticosteroid therapy, dyslipidemias, previous abdominal and/or pelvic surgery, collagenopathies, immunodeficiency syndromes. Pre-operative factors are: nutritional status (albumin and protein levels), hemoglobin level, nRT, localization of the tumor expressed in cm from the anal margin at rigid rectoscopy (“high” when located between 11 and 15 cm, “middle” between 6 and 10 cm, “low” up to 5 cm from the anal margin). Variants of surgical technique are: colorectal/colo-anal anastomosis type [straight colo-rectal (SCR), side-to-end (STE), end-to-side (ETS)], TME, diverting stoma. Post-operative factors are: surgical complications, need for re-intervention, blood transfusions, adjuvant chemotherapy (CTa), adjuvant radiotherapy (RTa), timing of closure of diverting stoma (earlier or later than 3 months after the index surgery).

### Statistical analysis

Data were analyzed using descriptive and inferential statistics. Categorical variables were expressed as absolute/relative frequencies. The impact of all risk factors was calculated using Fisher’s Exact Test for categorical variables and Mann–Whitney’s test for continuous variables. All *p* values reported were obtained by the 2-sided method, at the conventional significance level of 5%. Univariate and multivariate binary logistic regression was performed. Data were analyzed starting from September 2020 by R 4.0.2 (R Foundation for Statistical Computing, Vienna-A, http://www.R-project.org).

## Results

The sample included 153 consecutive patients with a median age of 66 years (range 34–91). Forty-one patients were affected by “low” rectal cancer, 74 by “middle” rectal cancer, 38 by “high” rectal cancer. Eighty (52.3%) patients were over 65 years of age. Sixty-three (41.2%) patients were female and 90 (58.8%) male. BMI was > 30 kg/m^2^ in 11 (7.2%) patients. The most common comorbidity was cardiovascular disease (56.9%), compared to dysmetabolic (diabetes 15%, dyslipidemia 21.6%), respiratory (8.5%), neurological (3.9%) and auto-immune (collagenopathies 2%) diseases; none of these factors were significant associated with LARS. Forty-six (30.1%) patients received long course nRT of which 24 patients had “low” rectal neoplasia, 18 patients had “middle” rectal neoplasia, 4 patients had “high” rectal neoplasia. All the patients underwent mechanical bowel preparation. One hundred and sixteen (76.8%) TMEs were performed, the predominantly colorectal anastomosis was SCR (94.8%), transanal mechanical (89.5%), and in 47.7% of cases (73 patients) a diverting stoma was performed. Sixty-four (43.2%) patients performed CTa and 3 (2%) patients performed RTa. All baseline patients’ characteristics are shown in Table 1. Complications rate was 21.6% (33 cases). All complications are summarized in Tables 2 and 3.

**Table 1:** Patients’ main characteristics at baseline

	Risk factors	*N*° patients/tot (%)
Anamnesis	Male gender	90/153 (58.8)
	Age > 65 years	80/153 (52.3)
	Smoking	25/153 (16.3)
	BMI > 30 kg/m^2^	11/153 (7.2)
	**Cardiovascular disease**	**87/153 (56.9)**
	Diabetes	23/153 (15.0)
	Neurological disorders	6/152 (3.9)
	COPD	13/153 (8.5)
	Corticosteroid therapy	2/153 (1.3)
	Dyslipidaemia	33/153 (21.6)
	Previous surgery	37/153 (24.2)
	Collagenopathies	3/153 (2.0)
	Immunodeficiency	0/153 (0.0)
Pre-operative data	Albumin level < 3.5 g/dl	0/148 (0.0)
	Protein level < 6.6 g/dl	40/136 (29.4)
	Hemoglobin < 12 g/dL	36/152 (23.7)
	nRT	46/153 (30.1)
	Localization of tumor	
	0–5 cm	41/153 (26.8)
	6–10 cm	74/153 (48.4)
	11–15 cm	38/153 (24.8)
	Bowel preparation	153/153 (100.0)
Surgical technique	Anastomosis	
	SCR	145/153 (94.8)
	STE	7/153 (4.6)
	ETS	1/153 (.7)
	TME	116/151 (76.8)
	Stoma	73/153 (47.7)
Post-operative data	Complications	33/153 (21.6)
	Re-intervention	8/145 (5.5)
	Blood transfusion	9/152 (5.9)
	CTa	64/148 (43.2)
	RTa	3/148 (2.0)
	Stoma closure (> 3 months)	61/73 (83.6)

Bold type indicates the most frequent anamnestic risk factor in the population

*BMI* body mass index, *COPD* chronic obstructive pulmonary disease, *nRT* neoadjuvant radiotherapy, *SCR* straight colo-rectal, *STE* side-to-end, *ETS* end-to-side, *TME* total mesorectal excision, *CTa* adjuvant chemotherapy, *RTa* adjuvant radiotherapy

**Table 2:** Classification of complications according to Clavien–Dindo

Clavien–Dindo I	Clavien–Dindo II	Clavien–Dindo III	Clavien–Dindo IVa	Clavien–Dindo V	Total
3/33 (9.1%)	22/33 (66.7%)	7/33 (21.2%)	1/33 (3.0%)	–	33/153 (21.6%)

**Table 3:** Complications, treatment and corresponding tumor localization

Complications	*n*	Treatment	Tumor localization
Anastomotic dehiscence	9	n°4 Stomas subsequently closed	n°1 High rectum, n° 3 middle rectum
		n°1 Suture of anastomosis	High rectum
		n°4 Conservative treatments	n°2 High rectum, n° 2 middle rectum
Bleeding	8	n°1 Surgical hemostasis	High rectum
		n°6 Blood transfusions	n°4 High rectum, n° 1 middle rectum, n° 1 low rectum
		n°1 Conservative treatment	Middle rectum
Rectovaginal fistula	2	n°2 Stomas subsequently closed	n°2 Low rectum
Intra-abdominal infections	5	n°5 Antibiotic therapies	n°1 High rectum, n° 3 middle rectum, n° 1 low rectum
Fever	5	n°5 Antibiotic therapies	n°2 Middle rectum, n° 3 low rectum
Pulmonary complications	2	n°2 Antibiotic therapies + oxygen-therapy	n°1 Middle rectum, n° 1 low rectum
Ileus	1	Fasting	Middle rectum
Linforrea	1	Conservative treatment	Middle rectum

The prevalence of overall LARS (major LARS + minor LARS) in our series was 35.9% (55/153 cases), minor 13.7% (21 cases) and major 22.2% (34 cases). In our study nRT and tumor localization resulted to be significantly associated with development of LARS. Association between nRT and overall/major LARS was significant (respectively *p* = 0.03 and 0.02), while the association with the minor form was not significant (*p* = 0.799). Distal localization of tumor was also significantly associated with LARS [overall LARS (*p* = 0.03), major LARS (*p* = 0.014), minor LARS (*p* = 0.365)]. All risk factors and the association with the development of minor, major LARS and overall (minor + major) LARS are summarized in Table 4.

**Table 4:** Distribution and analysis of possible LARS risk factors

Risk factor	*n* (%)	Minor LARS		Major LARS		Overall LARS (minor + major)	
		*n* (%)	*p*	*n* (%)	*p*	*n* (%)	*p*
Male gender	90 (58.8)	12 (13.3)	1.000	19 (21.1)	.698	31 (34.4)	.733
Age > 65 years	80 (52.3)	6 (7.5)	0.033	18 (22.5)	1.000	24 (30.0)	0.174
Smoking	25 (16.3)	5 (20.0)	.343	6 (24.0)	.796	11 (44.0)	.371
BMI > 30 kg/m^2^	11 (7.2)	3 (27.3)	.177	4 (36.4)	.263	7 (63.6)	.057
Cardiovascular disease	87 (56.9)	13 (14.9)	.645	19 (21.8)	1.000	32 (36.8)	.866
Previous surgery	37 (24.2)	3 (8.1)	.410	7 (18.9)	.656	10 (27.0)	.240
Protein level < 6.6 g/dl	40 (29.4)	5 (12.5)	1.000	8 (20.0)	.662	13 (32.5)	.696
Hemoglobin < 12 g/dl	36 (23.7)	3 (8.3)	.408	11 (30.6)	.179	14 (38.9)	.696
nRT	46 (30.1)	7 (15.2)	.799	18 (39.1)	**.002**	25 (54.3)	**.003**
Tumor localization							
0–5 cm	41 (26.8)	8 (19.5)	.365	16 (39.0)	**.014**	24 (58.5)	**.003**
6–10 cm	74 (48.4)	10 (13.5)		11 (14.9)		21 (28.4)	
11–15 cm	38 (24.8)	3 (7.9)		7 (18.4)		10 (26.3)	
Bowel preparation	153 (100.0)	21 (13.7)	–	34 (22.2)	–	55 (35.9)	–
Anastomosis							
SCR	145 (94.8)	20 (13.8)	1.000	31 (21.4)	.175	51 (35.2)	.410
STE	7 (4.6)	1 (14.3)		2 (28.6)		3 (42.9)	
ETS	1 (0.7)	0 (0.0)		1 (100.0)		1 (100.0)	
TME	116 (76.8)	18 (15.5)	.408	27 (23.3)	.347	45 (38.8)	.106
Stoma	73 (47.7)	11 (15.1)	.815	21 (28.8)	.080	32 (43.8)	.064
Complications	33 (21.6)	4 (12.1)	1.000	8 (24.2)	.814	12 (36.4)	1.000
Re-intervention	8 (5.5)	1 (12.5)	1.000	2 (25.0)	.670	3 (37.5)	1.000
Blood transfusion	9 (5.9)	1 (11.1)	1.000	1 (11.1)	.685	2 (22.2)	.492
CTa	64 (43.2)	8 (12.5)	.812	18 (28.1)	.238	26 (40.6)	.392
RTa	3 (2.0)	0 (0.0)	1.000	1 (33.3)	.546	1 (33.3)	1.000
Stoma closure (> 3 months)	61 (83.6)	9 (14.8)	.686	19 (31.1)	.324	28 (45.9)	.561

Bold type indicates statistically significant *p*-values

*BMI* body mass index, *nRT* neoadjuvant radiotherapy, *SCR* straight colo-rectal, *STE* side-to-end, *ETS* end-to-side, *TME* total mesorectal excision, *CTa* adjuvant chemotherapy, *RTa* adjuvant radiotherapy

### Univariate and multivariate analysis

On the univariate analysis, the nRT was a risk factor of overall LARS (major and minor) (OR 3.06, 95% CI 1.49- 6.26, *p* = 0.02), as well as the localization of tumor (*p* = 0.003). The localization of the tumor in “middle” and “high” rectum were protective factors compared to the localization in “low” rectum (respectively OR 0.28; 95% 0.13–0.63, *p* = 0.002 and OR 0.25; 95% CI 0.10–0.66, *p* = 0.005]. On the multivariate analysis, nRT was confirmed as an independent risk factor for the development of LARS (OR 2.18 CI 95% 1.00–4.78, *p* = 0.05), while the localization in “middle” and “high” rectum resulted as protective factors, if compared to the localization in “low” rectum (respectively OR 0.36, 95% CI 0.15–0.82, *p* = 0.015 and OR 0.36; 95% CI 0.13–1.00, *p* = 0.049) (Table 5).

**Table 5:** Univariate and multivariate analysis

Variable	Univariate analysis			Multivariate analysis		
	OR	95% CI	*p*	OR	95% CI	*p*
Gender (Male)	0.85	0.44–1.67	0.643	–	–	–
Age > 65 years	0.60	0.31–1.17	0.135	–	–	–
nRT	3.06	1.49–6.26	0.002	2.18	1.00–4.78	0.05
Tumor localization (cm from anal verge)			0.003			0.04
6–10 cm vs 0–5 cm	0.28	0.13–0.63	0.002	0.36	0.15–0.82	0.015
11–15 cm vs 0–5 cm	0.25	0.10–0.66	0.005	0.36	0.13–0.99	0.049
Outcome: overall LARS (minor + major)						

*OR* ods ratio, *CI* confidential interval, *nRT* neoadjuvant radiotherapy

## Discussion

Currently a multidisciplinary approach for the treatment of rectal cancer and the increase in “sphincter-saving” surgical techniques have significantly reduced the rate of definitive stoma, allowing the restoration of intestinal continuity in over 80% of patients after rectal resection. However, technical progress and improvement of oncological outcomes are associated with an important impact on patients’ QoL defined as LARS and surgical approach plays a significant role. It has been widely discussed over the years that laparoscopic surgery represents a valid alternative to the open approach both for short-term outcomes (smaller incisions, better post-operative pain control, shorter hospital stay, longer rapid resumption of daily activities), and long-term oncological outcomes (comparable oncological radicality and rates of loco-regional recurrence). In particular, recognized advantages of a minimally invasive approach derive from the better vision and, as a consequence, preservation of anatomical structures such as nerve plexus and anal sphincter function, all contributing to a reduction of surgical trauma [24–29]. At our Center, the minimally invasive approach represents the standard of care for rectal cancer.

To the best of our knowledge our study is currently the only one in the literature that analyzes the association between LARS and exclusively laparoscopic TMEs and the aim of the study was to estimate the prevalence of LARS at our Center and to define LARS risk factors in patients undergoing laparoscopic surgery for rectal cancer.

LARS rates after rectal surgery are quite heterogeneous, influenced by different post-operative evaluation strategies, different surgical approaches (open surgery, laparoscopic, robotic) and different rates of risk factors (radiotherapy, obesity, tumor site, etc.). Croese et al. in 2018 published a meta-analysis, in which they analyzed 11 articles, reporting variable prevalence of LARS from 17 to 65.4% [21]. We report a 35.9% rate of both minor and major LARS in a consecutive series of 153 laparoscopic resections, including long course nRT 30.1% rate.

Neoadjuvant radiotherapy/chemo-radiotherapy are considered among the main risk factors of LARS. In the study conducted by Hughes, the risk of major LARS was 20 times higher in patients who had previously undergone nCRT (*p* < 0.001) [30]. Bondeven et al. demonstrated that nRT was an independent risk factor for major LARS (OR 3.5; 95% CI 1.15–9.4) [13]. Peeters in 2005 demonstrated that neoadjuvant short-course radiotherapy followed by TME caused late side effects of intestinal dysfunction compared with surgery alone; equally in the Dutch Trial short-course nRT was a risk factor for major LARS (56% nRT vs 36% non-nRT; *p* < 0.01) [16]. In 2019 Sun showed that patients undergoing long-course nRT had a more severe form of LARS and the QoL was worse [17]. None of the previous mentioned series includes only laparoscopic TMEs. In our laparoscopic series long course nRT was performed in 46 patients (30.1%), more than half of whom (25 patients, 54.3%) developed LARS (*p* = 0.003).

Furthermore, there is a general consensus on the distance from anal verge as risk factors of LARS. In Battersby’s study, tumor localization within 6 cm of the anal margin was a high risk factor for severe LARS [12]. Bondeven, using post-operative Magnetic Resonance Imaging, evaluated what impact the height of the anastomosis had on post-operative bowel function: the risk of major LARS was 46% in patients with less than 4 cm of residual rectal stump compared to 10% in patients over 4 cm (*p* < 0.0001) [13]. Similar results were obtained by Ekkarat in cases of lower anastomosis, less than 5 cm from the anal margin [15]. Similarly in our study 58.5% of patients affected by “low” rectal cancer developed LARS. Bregendahl analyzed a series of 938 patients who underwent rectal resection between 2001 and 2007 in Denmark, performing 555 TME (59%): TME compared to PME (partial mesorectal excision) was significantly associated with the development of major LARS (OR 2.31; 95% CI 1.69–3.16) [14]. In our study, a total of 116 TMEs (76.8%) were performed. The association between TME and minor LARS (*p* = 0.408), major LARS (*p* = 0.347), overall LARS (*p* = 0.408) was not significant (*p* = 0.106). These data are probably due to the small size of the sample.

Also diverting stoma and delayed closure are associated with increased morbidity. Hughes in his 85 patients sample showed that stoma closure within 6 months was a protective factor for the development of LARS (OR 0.2; *p* < 0.01), while delayed closure increased the risk of developing LARS by 3.7 times [30]. In our series, a diverting stoma was performed in 73 patients (47.7%), in 61 of these (81.3%) it was closed after more than 3 months. The prevalence of overall LARS in patients with diverting stoma was 43.8% (*p* = 0.064), the prevalence of LARS in patients undergone delayed stoma closure (after 3 months) was 45.9% (*p* = 0.561) without statistical differences.

The type of colorectal anastomosis after resection also affects post-surgery morbidity, in particular intestinal function. According to the major randomized studies in the literature, the comparison between SCR (straight colo-rectal) anastomosis and J-pouch anastomosis shows that the creation of a new rectal reservoir reduces the frequency of defecation by 25–60% and varies the urgency from 0 to 100% [18]. In our study, the association between the type of anastomosis and the development of overall LARS was not significant (*p* = 0.409), since in almost all patients (94.8%) a SCR anastomosis was performed and therefore the groups of patients with different types of anastomosis were not comparable.

Finally among anamnestic risk factors obesity seems to represent a predictor of LARS. In the ROLARR trial published by Bolton, Univariate Multivariate Logistic Regression Models for Each Predictor of Interest showed that a 10-unit increase in BMI was associated with an increase of 2.456 (95% CI 0.942–6.348) in odds of major LARS [31]. In our series 11 patients had BMI > 30 kg/m^2^. Seven patients (63.6%) developed LARS, 3 of which reported the minor form and 4 the major form. BMI was not statistically associated with the development of overall LARS (*p* = 0.057), LARS minor (*p* = 0.177), LARS major (*p* = 0.263). The small size of the sample in our study may have affected the statistical significance of this data.

To date, several treatment options have been proposed for LARS, all mainly limited to symptom control. There is no well-defined standard protocol, but variable and patient-based. This includes antidiarrheal medications, anal plugs, biofeedback therapy, pelvic floor rehabilitation, colonic irrigation and nerve stimulation. Starting from the least invasive to the most invasive, these are used most frequently in combination, in order to obtain the best treatment for each patient. When all conservative treatments fail, a permanent stoma represents the ultimate option [23].

Main strengths of our work include sample homogeneity due to reduced patient recruitment time, low rate of missing data due to low patient loss rate at follow-up, and high response rate (almost all patients). Furthermore, to date, our study represents a novelty in the literature for its exclusively laparoscopic series. Patients were asked to answer the LARS Score questionnaire, referring to the first 12–18 months after surgery, in order to consider the typical time of LARS symptoms occurrence. We also performed a stratification of risk factors (anamnestic, pre-, intra- and post-operative), in order to evaluate the role of each on the prevalence of LARS. Finally, we included in the study patients who underwent surgery with only one type of surgical approach, to minimize the influence of the technique (open vs laparoscopic vs robotic) on the outcome. On the other hand, the retrospective analysis of a prospective database, the limited sample size and the inaccuracy of anastomotic height assessment represent the main limitations of our study.

Based on the aforementioned advantages of laparoscopy and on the etiology of the syndrome, it seems reasonable to expect a lower rate of LARS after laparoscopy compared with the open approach. However, the LARS rate in our series of laparoscopic resections remains high (35.9%), consistent with the open TME literature. Ultimately, this raises the hypothesis that other risk factors such as nRT, tumor location and anastomotic distance are more influent than the surgical technique.

Considering the high morbidity of rectal surgery, we therefore believe that in all Colorectal Surgery Centers it is necessary to provide a perioperative work-up, including evaluation questionnaires (e.g. LARS Score, Wexner Score, etc.), functional tests (e.g. pre-operative ano-rectal manometry) and rehabilitation programs [e.g. biofeedback, pelvic floor muscle training (PFMT), rectal balloon training (RBT) and trans-anal irrigation (TAI)], in order to select the most suitable treatment for each patient, as already proposed by Martellucci with a treatment algorithm in a multimodal approach [32]. Further studies are needed to compare LARS rate when performing different types of surgical approaches (open vs minimally invasive) for rectal cancer.

In conclusion LARS is a complex, multifactorial syndrome with high prevalence after rectal surgery. In our laparoscopic series, LARS rate resulted consistent with the open TME literature. Neoadjuvant radiotherapy and tumor localization resulted independent risk factors for LARS. Tumor localization in the “middle” and “high” rectum resulted a protective factor compared to the localization in “low” rectum.
